# Bis(3-carb­oxy-5-nitro­benzoato)bis­[2-(pyridin-4-yl)-1*H*-imidazo[4,5-*f*][1,10]phenanthroline]manganese(II)

**DOI:** 10.1107/S1600536811004569

**Published:** 2011-02-12

**Authors:** Hong-Bin Xu, Shuai Ma, Yu He

**Affiliations:** a China Science and Technology Exchange Center, Ministry of Science and Technology, Beijing 100045, People’s Republic of China; b College of Chemistry, Jilin Normal University, Siping 136000, People’s Republic of China

## Abstract

In the title compound, [Mn(C_8_H_4_NO_6_)_2_(C_18_H_11_N_5_)_2_], the Mn^II^ atom is six-coordinated by two *N*,*N*′-bidentate 6-(pyridin-4-yl)-5*H*-cyclo­penta­[*f*][1,10]phenanthroline (pcp) ligands and two carboxyl­ate O atoms from two monodentate 3-carb­oxy-5-nitro­benzoate anions in a distorted *cis*-MnO_2_N_4_ octa­hedral arrangement. Within the pcp ligands, the dihedral angles between the polycyclic skeletons and pendant pyridine rings are 6.2 (2) and 8.3 (2)°. In the crystal, mol­ecules are linked by O—H⋯N and N—H⋯O hydrogen bonds. Several aromatic π–π stacking inter­actions [shortest centroid–centroid separation = 3.516 (3) Å] are also observed.

## Related literature

For background to ligands based on 1,10-phenanthroline, see: Wang *et al.* (2010 ▶).
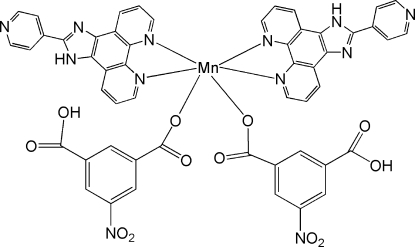


## Experimental

### 

#### Crystal data

[Mn(C_8_H_4_NO_6_)_2_(C_18_H_11_N_5_)_2_]
*M* *_r_* = 1069.82
Monoclinic, 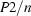

*a* = 21.791 (3) Å
*b* = 8.2215 (12) Å
*c* = 27.270 (4) Å
β = 111.767 (3)°
*V* = 4537.2 (12) Å^3^
*Z* = 4
Mo *K*α radiation
μ = 0.38 mm^−1^
*T* = 293 K
0.22 × 0.18 × 0.16 mm

#### Data collection

Bruker APEX CCD diffractometer
Absorption correction: multi-scan (*SADABS*; Sheldrick, 1996 ▶) *T*_min_ = 0.45, *T*_max_ = 0.69
22806 measured reflections
8037 independent reflections
3602 reflections with *I* > 2σ(*I*)
*R*_int_ = 0.105

#### Refinement

*R*[*F* ^2^ > 2σ(*F* ^2^)] = 0.065
*wR*(*F* ^2^) = 0.126
*S* = 0.95
8037 reflections
694 parameters
H-atom parameters constrained
Δρ_max_ = 0.29 e Å^−3^
Δρ_min_ = −0.24 e Å^−3^

### 

Data collection: *SMART* (Bruker, 1997 ▶); cell refinement: *SAINT* (Bruker, 1999 ▶); data reduction: *SAINT*; program(s) used to solve structure: *SHELXS97* (Sheldrick, 2008 ▶); program(s) used to refine structure: *SHELXL97* (Sheldrick, 2008 ▶); molecular graphics: *DIAMOND* (Brandenburg, 2006 ▶); software used to prepare material for publication: *SHELXL97*.

## Supplementary Material

Crystal structure: contains datablocks global, I. DOI: 10.1107/S1600536811004569/hb5800sup1.cif

Structure factors: contains datablocks I. DOI: 10.1107/S1600536811004569/hb5800Isup2.hkl

Additional supplementary materials:  crystallographic information; 3D view; checkCIF report

## Figures and Tables

**Table:** 

Mn1—O1	2.142 (3)
Mn1—O6	2.142 (3)
Mn1—N1	2.339 (4)
Mn1—N2	2.220 (3)
Mn1—N6	2.221 (4)
Mn1—N7	2.309 (4)

**Table:** 

N6—Mn1—N7	72.19 (14)
N2—Mn1—N1	72.42 (13)

**Table 2:** Hydrogen-bond geometry (Å, °)

*D*—H⋯*A*	*D*—H	H⋯*A*	*D*⋯*A*	*D*—H⋯*A*
O7—H9*A*⋯N10^i^	0.82	1.81	2.629 (5)	176
O4—H4⋯N5^ii^	0.82	1.82	2.636 (5)	173
N9—H7*A*⋯O2^iii^	0.86	1.94	2.789 (5)	171
N4—H4*A*⋯O5^iv^	0.86	1.89	2.745 (5)	171

Symmetry codes: (i) 
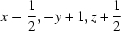
; (ii) 
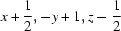
; (iii) 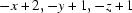; (iv) 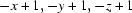.

**Fig. 1.**
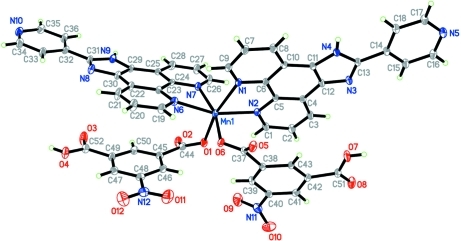
The asymmetric unit in (I) with isplacement ellipsoids drawn at the 30% probability level.

## supplementary crystallographic information

### Comment

The coordination complexes based on 1,10-phenanthroline-like ligands have received intense interests of chemists (Wang *et al.*, 2010). The (6-(pyridin-4-yl)-5H-cyclopenta[f][1,10]phenanthroline ligand (L), as a good candidate for N-donor ligand, has excellent coordinating ability. In this work, we selected 1,3-Hbdc ligand (1,3-Hbdc = 5-nitro-benzene-1-carboxylate-3-carboxylic acid) as a secondary ligand and L as a N-donor chelating ligand, generating a new molecular Mn^II^ complex, [Mn(L)_2_(1,3-Hbdc)_2_].

The central Mn^II^ atom is six-coordinated by four N atoms from two different L ligands, and two carboxylate O atoms from two different 1,3-Hbdc ligands in a distorted octahedral sphere. The O—H···N and N—H···O H-bonding interactions further stabilize the structure of (I).

### Experimental

A mixture of MnCl_2_^.^4H_2_O (0.5 mmol), 1,3-H_2_bdc (0.5 mmol) and L (0.5 mmol) in 1 ml distilled water was heated at 460 K in a Teflon-lined stainless steel autoclave for seven days. The reaction system was then slowly cooled to room temperature. Pale yellow blocks of (I) were collected from the final reaction system by filtration, washed several times with distilled water and dried in air at ambient temperature. Yield: 31% based on Mn(II).

### Refinement

All H atoms were positioned geometrically (C–H = 0.93 Å) and refined as riding, with U_iso_(H) = 1.2U_eq_(carrier).

### Crystal data

**Table:**

[Mn(C_8_H_4_NO_6_)_2_(C_18_H_11_N_5_)_2_]	*F*(000) = 2188
*M**_r_* = 1069.82	*D*_x_ = 1.566 Mg m^−^^3^
Monoclinic, *P*2/*n*	Mo *K*α radiation, λ = 0.71073 Å
Hall symbol: -P 2yac	Cell parameters from 8037 reflections
*a* = 21.791 (3) Å	θ = 1.5–25.1°
*b* = 8.2215 (12) Å	µ = 0.38 mm^−^^1^
*c* = 27.270 (4) Å	*T* = 293 K
β = 111.767 (3)°	Block, pale yellow
*V* = 4537.2 (12) Å^3^	0.22 × 0.18 × 0.16 mm
*Z* = 4	

### Data collection

**Table:**

Bruker APEX CCD diffractometer	8037 independent reflections
Radiation source: fine-focus sealed tube	3602 reflections with *I* > 2σ(*I*)
graphite	*R*_int_ = 0.105
φ and ω scans	θ_max_ = 25.1°, θ_min_ = 1.5°
Absorption correction: multi-scan (*SADABS*; Sheldrick, 1996)	*h* = −25→25
*T*_min_ = 0.45, *T*_max_ = 0.69	*k* = −9→9
22806 measured reflections	*l* = −32→31

### Refinement

**Table:**

Refinement on *F*^2^	Primary atom site location: structure-invariant direct methods
Least-squares matrix: full	Secondary atom site location: difference Fourier map
*R*[*F*^2^ > 2σ(*F*^2^)] = 0.065	Hydrogen site location: inferred from neighbouring sites
*wR*(*F*^2^) = 0.126	H-atom parameters constrained
*S* = 0.95	*w* = 1/[σ^2^(*F*_o_^2^) + (0.0333*P*)^2^] where *P* = (*F*_o_^2^ + 2*F*_c_^2^)/3
8037 reflections	(Δ/σ)_max_ = 0.001
694 parameters	Δρ_max_ = 0.29 e Å^−^^3^
0 restraints	Δρ_min_ = −0.24 e Å^−^^3^

### Special details

**Table:**

Geometry. All esds (except the esd in the dihedral angle between two l.s. planes) are estimated using the full covariance matrix. The cell esds are taken into account individually in the estimation of esds in distances, angles and torsion angles; correlations between esds in cell parameters are only used when they are defined by crystal symmetry. An approximate (isotropic) treatment of cell esds is used for estimating esds involving l.s. planes.
Refinement. Refinement of F^2^ against ALL reflections. The weighted R-factor wR and goodness of fit S are based on F^2^, conventional R-factors R are based on F, with F set to zero for negative F^2^. The threshold expression of F^2^ > 2sigma(F^2^) is used only for calculating R-factors(gt) etc. and is not relevant to the choice of reflections for refinement. R-factors based on F^2^ are statistically about twice as large as those based on F, and R- factors based on ALL data will be even larger.

### Fractional atomic coordinates and isotropic or equivalent isotropic displacement parameters (Å^2^)

**Table:**

	*x*	*y*	*z*	*U*_iso_*/*U*_eq_	
C1	0.7839 (2)	0.3222 (6)	0.62459 (18)	0.0436 (13)	
H1	0.8208	0.2673	0.6237	0.052*	
C2	0.7751 (2)	0.3328 (6)	0.67284 (18)	0.0517 (14)	
H2	0.8049	0.2840	0.7031	0.062*	
C3	0.7218 (3)	0.4161 (6)	0.67451 (18)	0.0493 (14)	
H3	0.7151	0.4250	0.7062	0.059*	
C4	0.6774 (2)	0.4880 (5)	0.62896 (18)	0.0370 (12)	
C5	0.6891 (2)	0.4700 (5)	0.58208 (17)	0.0341 (12)	
C6	0.6429 (2)	0.5362 (5)	0.53295 (18)	0.0340 (12)	
C7	0.5577 (3)	0.6533 (6)	0.43876 (19)	0.0543 (15)	
H7	0.5297	0.6911	0.4059	0.065*	
C8	0.5446 (2)	0.6859 (6)	0.48274 (19)	0.0478 (14)	
H8	0.5078	0.7473	0.4805	0.057*	
C9	0.6132 (3)	0.5630 (6)	0.44341 (19)	0.0479 (14)	
H9	0.6210	0.5401	0.4128	0.058*	
C10	0.5871 (2)	0.6262 (5)	0.53116 (18)	0.0350 (12)	
C11	0.5787 (2)	0.6459 (5)	0.58042 (17)	0.0348 (12)	
C12	0.6213 (2)	0.5789 (5)	0.62744 (18)	0.0386 (13)	
C13	0.5485 (3)	0.7048 (6)	0.64667 (18)	0.0405 (13)	
C14	0.5122 (2)	0.7806 (6)	0.67659 (18)	0.0411 (13)	
C15	0.5311 (2)	0.7421 (6)	0.72934 (19)	0.0513 (14)	
H15	0.5663	0.6719	0.7453	0.062*	
C16	0.4966 (3)	0.8096 (6)	0.7584 (2)	0.0570 (15)	
H16	0.5087	0.7800	0.7937	0.068*	
C17	0.4303 (2)	0.9544 (6)	0.6870 (2)	0.0531 (15)	
H17	0.3963	1.0289	0.6721	0.064*	
C18	0.4614 (2)	0.8892 (6)	0.65543 (19)	0.0475 (14)	
H18	0.4479	0.9189	0.6201	0.057*	
C19	0.7020 (3)	0.2939 (6)	0.3780 (2)	0.0560 (15)	
H19	0.6655	0.2432	0.3812	0.067*	
C20	0.7083 (3)	0.2884 (7)	0.3291 (2)	0.0684 (18)	
H20	0.6764	0.2379	0.3004	0.082*	
C21	0.7624 (3)	0.3590 (7)	0.32447 (19)	0.0704 (18)	
H21	0.7685	0.3539	0.2925	0.084*	
C22	0.8081 (3)	0.4382 (6)	0.36705 (19)	0.0450 (14)	
C23	0.7967 (2)	0.4395 (5)	0.41476 (18)	0.0361 (12)	
C24	0.8437 (2)	0.5203 (5)	0.46088 (18)	0.0347 (12)	
C25	0.9006 (2)	0.5974 (5)	0.45968 (19)	0.0366 (12)	
C26	0.8698 (3)	0.5916 (6)	0.5475 (2)	0.0563 (16)	
H26	0.8601	0.5896	0.5779	0.068*	
C27	0.9269 (3)	0.6722 (6)	0.54986 (19)	0.0594 (16)	
H27	0.9539	0.7235	0.5807	0.071*	
C28	0.9424 (2)	0.6740 (6)	0.50568 (19)	0.0545 (15)	
H28	0.9805	0.7260	0.5062	0.065*	
C29	0.9102 (2)	0.5912 (6)	0.41070 (19)	0.0389 (13)	
C30	0.8663 (3)	0.5148 (6)	0.36690 (19)	0.0453 (14)	
C31	0.9408 (3)	0.6143 (6)	0.3431 (2)	0.0475 (14)	
C32	0.9772 (3)	0.6646 (6)	0.3097 (2)	0.0489 (14)	
C33	0.9535 (3)	0.6121 (6)	0.2575 (2)	0.0639 (17)	
H33	0.9162	0.5463	0.2447	0.077*	
C34	0.9863 (3)	0.6595 (7)	0.2252 (2)	0.0756 (19)	
H34	0.9710	0.6210	0.1907	0.091*	
C35	1.0604 (3)	0.8106 (6)	0.2904 (2)	0.0579 (15)	
H35	1.0967	0.8800	0.3014	0.070*	
C36	1.0314 (3)	0.7674 (6)	0.3266 (2)	0.0561 (15)	
H36	1.0481	0.8067	0.3610	0.067*	
C37	0.6338 (3)	0.1061 (6)	0.50156 (19)	0.0403 (13)	
C38	0.6636 (2)	0.0379 (5)	0.55716 (18)	0.0349 (12)	
C39	0.7235 (2)	−0.0407 (5)	0.57444 (18)	0.0434 (13)	
H39	0.7468	−0.0535	0.5522	0.052*	
C40	0.7486 (2)	−0.1003 (5)	0.6254 (2)	0.0423 (13)	
C41	0.7181 (3)	−0.0741 (5)	0.66075 (19)	0.0472 (14)	
H41	0.7361	−0.1155	0.6949	0.057*	
C42	0.6608 (2)	0.0137 (6)	0.64488 (19)	0.0407 (13)	
C43	0.6323 (2)	0.0650 (5)	0.59284 (17)	0.0351 (12)	
H43	0.5917	0.1182	0.5815	0.042*	
C44	0.8736 (3)	0.1424 (6)	0.5129 (2)	0.0470 (14)	
C45	0.8530 (2)	0.0571 (6)	0.4602 (2)	0.0439 (14)	
C46	0.7958 (2)	−0.0331 (6)	0.4413 (2)	0.0482 (14)	
H46	0.7697	−0.0455	0.4614	0.058*	
C47	0.8125 (3)	−0.0839 (6)	0.3595 (2)	0.0495 (14)	
H47	0.7985	−0.1312	0.3262	0.059*	
C48	0.7772 (3)	−0.1052 (6)	0.3921 (2)	0.0477 (14)	
C49	0.8687 (3)	0.0087 (6)	0.3778 (2)	0.0494 (14)	
C50	0.8901 (2)	0.0752 (6)	0.42842 (19)	0.0470 (14)	
H50	0.9297	0.1325	0.4412	0.056*	
C51	0.6322 (3)	0.0621 (6)	0.6850 (2)	0.0479 (14)	
C52	0.9079 (3)	0.0441 (7)	0.3441 (2)	0.0551 (16)	
N1	0.65586 (18)	0.5075 (4)	0.48869 (15)	0.0378 (10)	
N2	0.74218 (19)	0.3867 (4)	0.58012 (14)	0.0347 (10)	
N3	0.6016 (2)	0.6158 (5)	0.66824 (14)	0.0434 (11)	
N4	0.53191 (18)	0.7276 (4)	0.59346 (14)	0.0393 (10)	
H4A	0.4989	0.7820	0.5725	0.047*	
N5	0.4470 (2)	0.9149 (5)	0.73788 (16)	0.0523 (12)	
N6	0.74414 (19)	0.3661 (5)	0.41993 (15)	0.0408 (10)	
N7	0.82803 (19)	0.5173 (5)	0.50504 (15)	0.0428 (11)	
N8	0.8854 (2)	0.5313 (5)	0.32436 (15)	0.0537 (12)	
N9	0.95818 (18)	0.6546 (4)	0.39486 (15)	0.0455 (11)	
H7A	0.9924	0.7090	0.4139	0.055*	
N10	1.0382 (2)	0.7566 (6)	0.24085 (18)	0.0602 (13)	
N11	0.8121 (2)	−0.1868 (6)	0.64354 (19)	0.0646 (14)	
N12	0.7165 (2)	−0.2004 (6)	0.3723 (2)	0.0667 (14)	
O1	0.82859 (16)	0.1734 (4)	0.53061 (12)	0.0502 (9)	
O2	0.93284 (18)	0.1821 (4)	0.53423 (13)	0.0639 (11)	
O3	0.9592 (2)	0.1171 (5)	0.36019 (15)	0.0900 (15)	
O4	0.87938 (17)	−0.0072 (4)	0.29559 (14)	0.0705 (11)	
H4	0.9027	0.0147	0.2788	0.106*	
O5	0.57270 (17)	0.1200 (4)	0.48219 (12)	0.0573 (10)	
O6	0.67361 (16)	0.1446 (3)	0.47948 (11)	0.0446 (9)	
O7	0.58037 (18)	0.1549 (4)	0.66617 (13)	0.0674 (11)	
H9A	0.5669	0.1773	0.6897	0.101*	
O8	0.65787 (18)	0.0239 (5)	0.73147 (14)	0.0726 (12)	
O9	0.8533 (2)	−0.1451 (6)	0.62548 (17)	0.1025 (16)	
O10	0.8222 (2)	−0.2911 (5)	0.67712 (18)	0.1011 (16)	
O11	0.6839 (2)	−0.2126 (5)	0.39982 (18)	0.1066 (16)	
O12	0.6987 (2)	−0.2588 (6)	0.32858 (19)	0.1047 (17)	
Mn1	0.74582 (4)	0.33502 (8)	0.50134 (3)	0.0390 (2)	

### Atomic displacement parameters (Å^2^)

**Table:**

	*U*^11^	*U*^22^	*U*^33^	*U*^12^	*U*^13^	*U*^23^
C1	0.033 (3)	0.056 (3)	0.039 (3)	0.007 (3)	0.011 (3)	−0.002 (3)
C2	0.047 (4)	0.071 (4)	0.033 (3)	0.018 (3)	0.011 (3)	0.007 (3)
C3	0.053 (4)	0.063 (4)	0.034 (3)	0.009 (3)	0.019 (3)	−0.003 (3)
C4	0.040 (3)	0.039 (3)	0.033 (3)	0.002 (3)	0.014 (3)	−0.001 (3)
C5	0.032 (3)	0.037 (3)	0.036 (3)	−0.004 (3)	0.016 (2)	0.000 (2)
C6	0.033 (3)	0.036 (3)	0.035 (3)	−0.001 (3)	0.016 (2)	0.000 (2)
C7	0.052 (4)	0.070 (4)	0.044 (3)	0.020 (3)	0.021 (3)	0.021 (3)
C8	0.037 (3)	0.059 (4)	0.050 (3)	0.009 (3)	0.019 (3)	0.005 (3)
C9	0.060 (4)	0.054 (4)	0.033 (3)	0.008 (3)	0.021 (3)	0.007 (3)
C10	0.034 (3)	0.038 (3)	0.034 (3)	−0.003 (3)	0.013 (2)	0.002 (3)
C11	0.029 (3)	0.040 (3)	0.036 (3)	−0.001 (3)	0.013 (2)	−0.006 (3)
C12	0.038 (3)	0.047 (3)	0.032 (3)	0.003 (3)	0.014 (3)	−0.006 (3)
C13	0.048 (4)	0.047 (3)	0.032 (3)	0.000 (3)	0.021 (3)	−0.004 (3)
C14	0.034 (3)	0.051 (3)	0.039 (3)	−0.003 (3)	0.015 (3)	−0.007 (3)
C15	0.050 (4)	0.068 (4)	0.039 (3)	0.011 (3)	0.021 (3)	0.002 (3)
C16	0.056 (4)	0.076 (4)	0.043 (3)	−0.001 (4)	0.024 (3)	−0.001 (3)
C17	0.038 (4)	0.067 (4)	0.059 (4)	0.003 (3)	0.023 (3)	−0.004 (3)
C18	0.042 (4)	0.058 (4)	0.047 (3)	−0.005 (3)	0.021 (3)	−0.010 (3)
C19	0.047 (4)	0.070 (4)	0.054 (4)	−0.013 (3)	0.023 (3)	−0.004 (3)
C20	0.061 (4)	0.099 (5)	0.040 (4)	−0.031 (4)	0.013 (3)	−0.012 (3)
C21	0.075 (5)	0.108 (5)	0.032 (3)	−0.030 (4)	0.026 (3)	−0.006 (3)
C22	0.047 (4)	0.056 (4)	0.036 (3)	−0.008 (3)	0.019 (3)	0.003 (3)
C23	0.028 (3)	0.048 (3)	0.034 (3)	0.000 (3)	0.013 (2)	0.004 (3)
C24	0.029 (3)	0.038 (3)	0.039 (3)	0.004 (3)	0.015 (2)	0.008 (3)
C25	0.031 (3)	0.038 (3)	0.043 (3)	0.002 (3)	0.016 (3)	0.006 (3)
C26	0.054 (4)	0.078 (4)	0.046 (4)	−0.017 (3)	0.028 (3)	−0.009 (3)
C27	0.058 (4)	0.079 (4)	0.042 (3)	−0.025 (4)	0.019 (3)	−0.013 (3)
C28	0.040 (3)	0.069 (4)	0.055 (4)	−0.019 (3)	0.018 (3)	−0.001 (3)
C29	0.034 (3)	0.051 (3)	0.041 (3)	0.001 (3)	0.024 (3)	0.010 (3)
C30	0.045 (4)	0.059 (4)	0.039 (3)	−0.005 (3)	0.022 (3)	0.006 (3)
C31	0.051 (4)	0.058 (4)	0.044 (4)	0.005 (3)	0.028 (3)	0.009 (3)
C32	0.046 (4)	0.063 (4)	0.048 (4)	0.008 (3)	0.028 (3)	0.019 (3)
C33	0.076 (5)	0.073 (4)	0.057 (4)	−0.014 (3)	0.041 (4)	0.000 (3)
C34	0.098 (6)	0.081 (5)	0.068 (4)	−0.012 (4)	0.055 (4)	0.001 (4)
C35	0.039 (3)	0.079 (4)	0.058 (4)	0.003 (3)	0.021 (3)	0.015 (4)
C36	0.043 (4)	0.083 (4)	0.048 (3)	0.007 (3)	0.024 (3)	0.018 (3)
C37	0.043 (4)	0.039 (3)	0.043 (3)	0.004 (3)	0.021 (3)	−0.001 (3)
C38	0.028 (3)	0.039 (3)	0.038 (3)	0.000 (3)	0.013 (2)	−0.001 (2)
C39	0.040 (3)	0.055 (4)	0.039 (3)	0.000 (3)	0.019 (3)	−0.008 (3)
C40	0.035 (3)	0.041 (3)	0.053 (4)	0.007 (3)	0.019 (3)	−0.002 (3)
C41	0.052 (4)	0.044 (3)	0.042 (3)	−0.007 (3)	0.013 (3)	0.002 (3)
C42	0.040 (3)	0.046 (3)	0.039 (3)	−0.003 (3)	0.019 (3)	0.002 (3)
C43	0.027 (3)	0.041 (3)	0.041 (3)	−0.002 (2)	0.016 (2)	−0.001 (3)
C44	0.040 (4)	0.052 (4)	0.051 (4)	0.008 (3)	0.020 (3)	0.011 (3)
C45	0.032 (3)	0.046 (3)	0.058 (4)	0.005 (3)	0.022 (3)	0.006 (3)
C46	0.045 (4)	0.051 (4)	0.055 (4)	0.011 (3)	0.026 (3)	0.011 (3)
C47	0.044 (4)	0.054 (4)	0.054 (4)	0.000 (3)	0.023 (3)	−0.001 (3)
C48	0.031 (3)	0.046 (3)	0.064 (4)	−0.006 (3)	0.015 (3)	0.004 (3)
C49	0.051 (4)	0.054 (4)	0.050 (4)	−0.005 (3)	0.027 (3)	0.002 (3)
C50	0.032 (3)	0.059 (4)	0.052 (4)	−0.005 (3)	0.019 (3)	0.001 (3)
C51	0.049 (4)	0.057 (4)	0.044 (4)	−0.005 (3)	0.024 (3)	−0.006 (3)
C52	0.054 (4)	0.067 (4)	0.048 (4)	−0.006 (3)	0.023 (3)	−0.003 (3)
N1	0.038 (3)	0.046 (3)	0.034 (2)	0.002 (2)	0.019 (2)	0.003 (2)
N2	0.029 (2)	0.043 (3)	0.032 (2)	0.001 (2)	0.0108 (19)	−0.001 (2)
N3	0.039 (3)	0.055 (3)	0.039 (3)	0.007 (2)	0.019 (2)	−0.006 (2)
N4	0.038 (3)	0.045 (3)	0.038 (3)	0.007 (2)	0.018 (2)	0.001 (2)
N5	0.048 (3)	0.069 (3)	0.042 (3)	−0.003 (3)	0.020 (2)	−0.012 (3)
N6	0.027 (2)	0.052 (3)	0.042 (3)	−0.009 (2)	0.011 (2)	−0.003 (2)
N7	0.043 (3)	0.053 (3)	0.038 (3)	−0.006 (2)	0.023 (2)	−0.003 (2)
N8	0.050 (3)	0.077 (3)	0.041 (3)	−0.010 (3)	0.025 (2)	0.004 (2)
N9	0.036 (3)	0.060 (3)	0.046 (3)	−0.005 (2)	0.022 (2)	0.005 (2)
N10	0.056 (3)	0.075 (4)	0.060 (3)	0.007 (3)	0.034 (3)	0.016 (3)
N11	0.054 (4)	0.074 (4)	0.060 (4)	0.011 (3)	0.014 (3)	−0.009 (3)
N12	0.050 (4)	0.064 (4)	0.088 (4)	0.003 (3)	0.028 (3)	0.008 (3)
O1	0.044 (2)	0.063 (2)	0.054 (2)	0.011 (2)	0.0301 (18)	0.0069 (19)
O2	0.046 (3)	0.093 (3)	0.058 (2)	−0.007 (2)	0.027 (2)	−0.009 (2)
O3	0.073 (3)	0.145 (4)	0.066 (3)	−0.048 (3)	0.042 (2)	−0.026 (3)
O4	0.070 (3)	0.102 (3)	0.050 (2)	−0.022 (3)	0.034 (2)	−0.012 (2)
O5	0.038 (2)	0.088 (3)	0.048 (2)	0.011 (2)	0.0190 (18)	0.016 (2)
O6	0.050 (2)	0.052 (2)	0.040 (2)	−0.0106 (19)	0.0256 (17)	−0.0011 (17)
O7	0.065 (3)	0.093 (3)	0.060 (2)	0.021 (3)	0.041 (2)	0.000 (2)
O8	0.078 (3)	0.106 (3)	0.039 (2)	0.004 (3)	0.028 (2)	0.000 (2)
O9	0.065 (3)	0.146 (4)	0.107 (4)	0.043 (3)	0.044 (3)	0.016 (3)
O10	0.075 (4)	0.083 (3)	0.119 (4)	0.007 (3)	0.005 (3)	0.028 (3)
O11	0.079 (4)	0.133 (4)	0.120 (4)	−0.049 (3)	0.052 (3)	−0.008 (3)
O12	0.069 (3)	0.133 (4)	0.108 (4)	−0.027 (3)	0.028 (3)	−0.042 (3)
Mn1	0.0346 (5)	0.0506 (5)	0.0381 (4)	−0.0011 (4)	0.0207 (3)	0.0009 (4)

### Geometric parameters (Å, °)

**Table:**

C1—N2	1.327 (5)	C30—N8	1.378 (5)
C1—C2	1.401 (5)	C31—N8	1.313 (6)
C1—H1	0.9300	C31—N9	1.359 (5)
C2—C3	1.363 (6)	C31—C32	1.473 (6)
C2—H2	0.9300	C32—C36	1.384 (6)
C3—C4	1.391 (6)	C32—C33	1.390 (6)
C3—H3	0.9300	C33—C34	1.381 (6)
C4—C5	1.401 (5)	C33—H33	0.9300
C4—C12	1.421 (6)	C34—N10	1.320 (6)
C5—N2	1.361 (5)	C34—H34	0.9300
C5—C6	1.451 (6)	C35—N10	1.330 (6)
C6—N1	1.358 (5)	C35—C36	1.400 (6)
C6—C10	1.410 (6)	C35—H35	0.9300
C7—C8	1.358 (6)	C36—H36	0.9300
C7—C9	1.385 (6)	C37—O5	1.242 (5)
C7—H7	0.9300	C37—O6	1.266 (5)
C8—C10	1.391 (6)	C37—C38	1.518 (6)
C8—H8	0.9300	C38—C39	1.374 (6)
C9—N1	1.321 (5)	C38—C43	1.398 (5)
C9—H9	0.9300	C39—C40	1.380 (6)
C10—C11	1.430 (6)	C39—H39	0.9300
C11—N4	1.373 (5)	C40—C41	1.377 (6)
C11—C12	1.388 (6)	C40—N11	1.469 (6)
C12—N3	1.367 (5)	C41—C42	1.366 (6)
C13—N3	1.311 (5)	C41—H41	0.9300
C13—N4	1.372 (5)	C42—C43	1.387 (6)
C13—C14	1.469 (6)	C42—C51	1.500 (6)
C14—C18	1.371 (6)	C43—H43	0.9300
C14—C15	1.378 (6)	C44—O2	1.247 (5)
C15—C16	1.393 (6)	C44—O1	1.269 (5)
C15—H15	0.9300	C44—C45	1.510 (6)
C16—N5	1.335 (6)	C45—C46	1.378 (6)
C16—H16	0.9300	C45—C50	1.395 (6)
C17—N5	1.337 (5)	C46—C48	1.382 (6)
C17—C18	1.386 (6)	C46—H46	0.9300
C17—H17	0.9300	C47—C49	1.369 (6)
C18—H18	0.9300	C47—C48	1.385 (6)
C19—N6	1.313 (5)	C47—H47	0.9300
C19—C20	1.390 (6)	C48—N12	1.458 (6)
C19—H19	0.9300	C49—C50	1.394 (6)
C20—C21	1.361 (7)	C49—C52	1.499 (6)
C20—H20	0.9300	C50—H50	0.9300
C21—C22	1.381 (6)	C51—O8	1.221 (5)
C21—H21	0.9300	C51—O7	1.300 (6)
C22—C23	1.413 (5)	C52—O3	1.199 (6)
C22—C30	1.416 (6)	C52—O4	1.305 (6)
C23—N6	1.347 (5)	Mn1—O1	2.142 (3)
C23—C24	1.455 (6)	Mn1—O6	2.142 (3)
C24—N7	1.368 (5)	Mn1—N1	2.339 (4)
C24—C25	1.404 (6)	Mn1—N2	2.220 (3)
C25—C28	1.397 (6)	Mn1—N6	2.221 (4)
C25—C29	1.428 (6)	Mn1—N7	2.309 (4)
C26—N7	1.326 (5)	N4—H4A	0.8600
C26—C27	1.391 (6)	N9—H7A	0.8600
C26—H26	0.9300	N11—O10	1.214 (5)
C27—C28	1.366 (6)	N11—O9	1.222 (5)
C27—H27	0.9300	N12—O12	1.208 (5)
C28—H28	0.9300	N12—O11	1.214 (5)
C29—N9	1.373 (5)	O4—H4	0.8200
C29—C30	1.375 (6)	O7—H9A	0.8200
			
N2—C1—C2	123.2 (4)	N10—C35—C36	122.8 (5)
N2—C1—H1	118.4	N10—C35—H35	118.6
C2—C1—H1	118.4	C36—C35—H35	118.6
C3—C2—C1	118.5 (4)	C32—C36—C35	118.2 (5)
C3—C2—H2	120.7	C32—C36—H36	120.9
C1—C2—H2	120.7	C35—C36—H36	120.9
C2—C3—C4	120.1 (5)	O5—C37—O6	126.2 (5)
C2—C3—H3	119.9	O5—C37—C38	116.9 (4)
C4—C3—H3	119.9	O6—C37—C38	116.9 (5)
C3—C4—C5	117.9 (5)	C39—C38—C43	118.9 (4)
C3—C4—C12	123.7 (4)	C39—C38—C37	121.1 (4)
C5—C4—C12	118.3 (4)	C43—C38—C37	119.9 (4)
N2—C5—C4	122.2 (4)	C38—C39—C40	118.9 (4)
N2—C5—C6	117.5 (4)	C38—C39—H39	120.5
C4—C5—C6	120.3 (4)	C40—C39—H39	120.5
N1—C6—C10	121.4 (4)	C41—C40—C39	122.4 (5)
N1—C6—C5	117.0 (4)	C41—C40—N11	118.6 (5)
C10—C6—C5	121.6 (4)	C39—C40—N11	118.9 (5)
C8—C7—C9	119.2 (5)	C42—C41—C40	118.9 (5)
C8—C7—H7	120.4	C42—C41—H41	120.6
C9—C7—H7	120.4	C40—C41—H41	120.6
C7—C8—C10	119.0 (5)	C41—C42—C43	119.7 (4)
C7—C8—H8	120.5	C41—C42—C51	119.2 (5)
C10—C8—H8	120.5	C43—C42—C51	121.1 (5)
N1—C9—C7	124.0 (5)	C42—C43—C38	121.0 (5)
N1—C9—H9	118.0	C42—C43—H43	119.5
C7—C9—H9	118.0	C38—C43—H43	119.5
C8—C10—C6	118.7 (4)	O2—C44—O1	125.7 (5)
C8—C10—C11	125.5 (5)	O2—C44—C45	117.1 (5)
C6—C10—C11	115.9 (4)	O1—C44—C45	117.2 (5)
N4—C11—C12	105.2 (4)	C46—C45—C50	118.5 (5)
N4—C11—C10	131.6 (4)	C46—C45—C44	121.0 (5)
C12—C11—C10	123.2 (4)	C50—C45—C44	120.5 (5)
N3—C12—C11	111.1 (4)	C45—C46—C48	119.6 (5)
N3—C12—C4	128.3 (5)	C45—C46—H46	120.2
C11—C12—C4	120.6 (4)	C48—C46—H46	120.2
N3—C13—N4	113.2 (4)	C49—C47—C48	118.1 (5)
N3—C13—C14	123.8 (4)	C49—C47—H47	121.0
N4—C13—C14	123.0 (5)	C48—C47—H47	121.0
C18—C14—C15	118.1 (4)	C46—C48—C47	122.4 (5)
C18—C14—C13	124.0 (4)	C46—C48—N12	119.3 (5)
C15—C14—C13	117.9 (5)	C47—C48—N12	118.2 (5)
C14—C15—C16	119.2 (5)	C47—C49—C50	120.3 (5)
C14—C15—H15	120.4	C47—C49—C52	121.4 (5)
C16—C15—H15	120.4	C50—C49—C52	118.3 (5)
N5—C16—C15	122.8 (5)	C49—C50—C45	121.0 (5)
N5—C16—H16	118.6	C49—C50—H50	119.5
C15—C16—H16	118.6	C45—C50—H50	119.5
N5—C17—C18	122.8 (5)	O8—C51—O7	123.9 (5)
N5—C17—H17	118.6	O8—C51—C42	122.3 (5)
C18—C17—H17	118.6	O7—C51—C42	113.7 (5)
C14—C18—C17	119.7 (5)	O3—C52—O4	124.2 (5)
C14—C18—H18	120.2	O3—C52—C49	122.6 (5)
C17—C18—H18	120.2	O4—C52—C49	113.2 (5)
N6—C19—C20	124.4 (5)	C9—N1—C6	117.7 (4)
N6—C19—H19	117.8	C9—N1—Mn1	127.4 (3)
C20—C19—H19	117.8	C6—N1—Mn1	114.4 (3)
C21—C20—C19	118.1 (5)	C1—N2—C5	118.0 (4)
C21—C20—H20	120.9	C1—N2—Mn1	123.3 (3)
C19—C20—H20	120.9	C5—N2—Mn1	118.0 (3)
C20—C21—C22	120.2 (5)	C13—N3—C12	104.5 (4)
C20—C21—H21	119.9	C13—N4—C11	106.1 (4)
C22—C21—H21	119.9	C13—N4—H4A	126.9
C21—C22—C23	117.3 (5)	C11—N4—H4A	126.9
C21—C22—C30	125.1 (5)	C16—N5—C17	117.4 (4)
C23—C22—C30	117.6 (5)	C19—N6—C23	117.3 (4)
N6—C23—C22	122.7 (4)	C19—N6—Mn1	124.5 (3)
N6—C23—C24	117.9 (4)	C23—N6—Mn1	117.2 (3)
C22—C23—C24	119.4 (4)	C26—N7—C24	116.4 (4)
N7—C24—C25	122.6 (4)	C26—N7—Mn1	127.4 (3)
N7—C24—C23	115.3 (4)	C24—N7—Mn1	114.6 (3)
C25—C24—C23	122.1 (4)	C31—N8—C30	104.1 (4)
C28—C25—C24	118.0 (4)	C31—N9—C29	106.4 (4)
C28—C25—C29	125.7 (5)	C31—N9—H7A	126.8
C24—C25—C29	116.3 (4)	C29—N9—H7A	126.8
N7—C26—C27	125.0 (5)	C34—N10—C35	118.4 (5)
N7—C26—H26	117.5	O10—N11—O9	123.2 (6)
C27—C26—H26	117.5	O10—N11—C40	118.7 (5)
C28—C27—C26	118.2 (5)	O9—N11—C40	118.0 (5)
C28—C27—H27	120.9	O12—N12—O11	122.1 (6)
C26—C27—H27	120.9	O12—N12—C48	119.6 (5)
C27—C28—C25	119.7 (5)	O11—N12—C48	118.2 (6)
C27—C28—H28	120.1	C44—O1—Mn1	130.2 (3)
C25—C28—H28	120.1	C52—O4—H4	109.5
N9—C29—C30	105.5 (4)	C37—O6—Mn1	127.8 (3)
N9—C29—C25	132.3 (5)	C51—O7—H9A	109.5
C30—C29—C25	122.2 (4)	O6—Mn1—O1	94.61 (12)
C29—C30—N8	110.8 (5)	O6—Mn1—N2	97.07 (12)
C29—C30—C22	122.5 (4)	O1—Mn1—N2	94.91 (13)
N8—C30—C22	126.6 (5)	O6—Mn1—N6	93.88 (13)
N8—C31—N9	113.2 (4)	O1—Mn1—N6	98.23 (12)
N8—C31—C32	122.2 (5)	N2—Mn1—N6	162.14 (13)
N9—C31—C32	124.5 (5)	O6—Mn1—N7	164.73 (12)
C36—C32—C33	118.5 (5)	O1—Mn1—N7	81.61 (13)
C36—C32—C31	123.7 (5)	N2—Mn1—N7	97.99 (13)
C33—C32—C31	117.6 (5)	N6—Mn1—N7	72.19 (14)
C34—C33—C32	118.8 (5)	O6—Mn1—N1	85.01 (13)
C34—C33—H33	120.6	O1—Mn1—N1	167.13 (12)
C32—C33—H33	120.6	N2—Mn1—N1	72.42 (13)
N10—C34—C33	123.3 (6)	N6—Mn1—N1	94.62 (13)
N10—C34—H34	118.4	N7—Mn1—N1	101.96 (12)
C33—C34—H34	118.4		

### Hydrogen-bond geometry (Å, °)

**Table:**

*D*—H···*A*	*D*—H	H···*A*	*D*···*A*	*D*—H···*A*
O7—H9A···N10^i^	0.82	1.81	2.629 (5)	176
O4—H4···N5^ii^	0.82	1.82	2.636 (5)	173
N9—H7A···O2^iii^	0.86	1.94	2.789 (5)	171
N4—H4A···O5^iv^	0.86	1.89	2.745 (5)	171

Symmetry codes: (i) *x*−1/2, −*y*+1, *z*+1/2; (ii) *x*+1/2, −*y*+1, *z*−1/2; (iii) −*x*+2, −*y*+1, −*z*+1; (iv) −*x*+1, −*y*+1, −*z*+1.
